# Cryptography-Based Medical Signal Securing Using Improved Variation Mode Decomposition with Machine Learning Techniques

**DOI:** 10.1155/2022/7307552

**Published:** 2022-09-12

**Authors:** Piyush Shukla, Oluwatobi Akanbi, Asakipaam Simon Atuah, Amer Aljaedi, Mohamed Bouye, Shakti Sharma

**Affiliations:** ^1^UIT-RGPV, Bhopal, India; ^2^Computer Science Department, University of Colorado, Colorado Springs, CO 80918, USA; ^3^Department of Telecommunication Engineering, KNUST (Kwame Nkrumah University of Science and Technology), Ghana; ^4^College of Computing and Information Technology, University of Tabuk, Tabuk 71491, Saudi Arabia; ^5^Department of Mathematics, College of Science, King Khalid University, Abha, Saudi Arabia; ^6^School of Computer Science Engineering & Technology, Bennett University, India

## Abstract

There is no question about the value that digital signal processing brings to the area of biomedical research. DSP processors are used to sample and process the analog inputs that are received from a human organ. These inputs come from the organ itself. DSP processors, because of their multidimensional data processing nature, are the electrical components that take up the greatest space and use the most power. In this age of digital technology and electronic gizmos, portable biomedical devices represent an essential step forward in technological advancement. Electrocardiogram (ECG) units are among the most common types of biomedical equipment, and their functions are absolutely necessary to the process of saving human life. In the latter part of the 1990s, portable electrocardiogram (ECG) devices began to appear on the market, and research into their signal processing and electronics design capabilities continues today. System-on-chip (SoC) design refers to the process through which the separate computing components of a DSP unit are combined onto a single chip in order to achieve greater power and space efficiency. In the design of biomedical DSP devices, this body of research presents a number of different solutions for reducing power consumption and space requirements. Using serial or parallel data buses, which are often the region that consumes the most power, it is possible to send data between the system-on-chip (SoC) and other components. To cut down on the number of needless switching operations that take place during data transmission, a hybrid solution that makes use of the shift invert bus encoding scheme has been developed. Using a phase-encoded shift invert bus encoding approach, which embeds the two-bit indication lines into a single-bit encoded line, is one way to solve the issue of having two distinct indicator bits. This method reduces the problem. The PESHINV approach is compared to the SHINV method that already exists, and the comparison reveals that the suggested PESHINV method reduces the total power consumption of the encoding circuit by around 30 percent. The computing unit of the DSP processor is the target of further optimization efforts. Virtually, all signal processing methods need memory and multiplier circuits to function properly.

## 1. Introduction

It should come as no surprise that video-based heart rate monitoring is becoming more popular [1], given the tremendous expansion of remote medical monitoring in recent years. In most instances, the signals for photoplethysmography and ballistocardiography are calculated by utilizing video pictures that were recorded during the process. These images were captured on a video camera. In order for them to work properly, they need to estimate either tiny color changes or stiff head and facial movements [2]. In the field of biomedical engineering, remote health monitoring is a notion that is still very new. When physiological indicators could be assessed using a digital camera, a major acceleration in the development of remote sensing technologies occurred [3]. The researchers were able to extract BCG signals by taking advantage of the involuntary head movement that was brought on by an increase in cerebral blood flow. As a direct consequence of this, the BCG signals were retrieved from the video of the face of a person. As a result of the contraction of the left ventricle, blood is forced across the aortic arch at a high rate. At the conclusion of each cycle of circulation, the carotid arteries transport blood back to the brain and the spinal column [4] because of their compact size, portability, comfort, and reasonable price. In addition, photoplethysmographic (PPG) signals are used in a large number of other applications outside those mentioned above. The results of some early study suggest that it would be able to use PPG signals to determine the rates of respiration, heart rate, and blood pressure. PPG signals received from the wrist are often utilized in athletic competition to monitor a variety of vital indicators, including heart rate (HR). The PPG device is advantageous for usage in home-based healthcare systems because of its user-friendliness, portability, comfort, and cost-efficiency. The PPG signal that is derived from pulse oximetry is one of the most promising possibilities in terms of physiological monitoring and omnipresent healthcare [5]. In this particular application, photoplethysmography, often known as PPG, has been shown to be quite successful [6]. Changes in the microvascular blood volume may be determined by the use of the optical approach. The ability of a tissue to reflect and transmit light, as stated by Beer's law, is what determines a tissue's capacity to draw blood, according to Lambert's law. This phenomena can be photographed with the commercial camera that is present on the majority of recent smartphones [7], despite the fact that it is difficult to notice with the human eye. The development of noninvasive cardiac monitoring techniques that utilize methods for detecting the PPG signal in both transmitted and reflected modes is approaching completion and will soon be used in many applications. PPG signal measurement is now concentrated on the transmission method, which detects signals at the fingertip. This mode is considered the state of the art in the field.

## 2. Literature Survey

Yu Rong et al. developed a technique for measuring distant plethysmographic signals that makes use of an inexpensive camera and the light that is already present in the environment [8]. When compared to the other two channels, the green channel (which includes red, green, and blue information) contains the greatest plethysmographic data (red, green, and blue). Heart rate was determined by Chunlei Wu and colleagues with the use of digital color footage that was acquired through a camera [9]. Utilizing a time-varying intensity signal generator makes it feasible to create a time-varying intensity signal from the intensity variance of face pixels. This may be done in a number of ways. The Viola–Jones face detector was used to successfully determine the identity of the suspect [10]. This method was used in order to get the face's pixel data. Throughout the whole of the experiment, it monitored the subject's face and recorded its movements. By performing a spatial average on the face pixels and then comparing these new signals to the original signals, the scientists were able to derive temporal signals with red, green, and blue intensity variations [11]. They were able to perfect their technique by applying temporal filtering to the PPG signal, which is what Chen Wang and his colleagues did. In order to make it easier to pick the region of interest (ROI), the authors shrunk the full face ROI to 60 percent of its original width and therefore limited the number of options available [12]. They were able to accomplish this feat because they were able to disregard pixels that were unrelated to the face [13]. Ningqi Luo et al. suggested a PPG-based heart rate measuring system that measures heart rate by using the green spectrum of an RGB camera [14]. Face detection was accomplished by applying a discriminative response map to the skin area below a person's eyes on the lower half of their face [15]. This region is situated on the bottom half of a person's face. As a consequence of this, we made use of KLT feature monitoring in order to monitor the return on investment over the course of time [16]. We were able to successfully eliminate motion artifacts from the green spectrum data by using a neural network. Because of this, we were able to totally automate the process of measuring the heart rate of patients. They were able to get the results they wanted by combining a nonrigid motion removal technique with a normalized least mean square adaptive filter. This allowed them to account for the effects of motion. According to the findings of their investigation, Lam et al. discovered that achieving equivalent results utilizing green spectrum data from an RGB camera was possible. The authors of this research used BSS to gather data after separating numerous green spectrum signals from random patches and merging them. The signals were obtained separately. PPGs were computed by Na Hye Kim et al. by conducting an analysis of the red and green spectra, respectively, of the red and green filters of the RGB camera [17]. The PPG signal was computed by the authors using an adaptive green and red differentiation function that they had designed themselves. The idea that chrominance characteristics on the face may be utilized to determine heart rate was recently introduced by researchers Jeremy Speth et al. [18]. An adaptive matrix calculation approach was developed by the authors of this work [19] in order to estimate the PPG signal from the chrominance aspects of the data used in this investigation. This method was put to the test in this work.

The PPG signal is modulated in a number of different ways by the breathing process. Modulations such as pulse frequency modulation, pulse amplitude modulation, and baseline modulation are included in this category. Chenglong Ye et al. assessed the respiratory rate by using a three-way average of three different respiratory rates (RRs) that were generated from three different changes in PPG that were caused by breathing [20]. In order to calculate the patient's respiration rate, they created the Lazaro algorithm. In addition, the RR and HR were created by Nakajima and his colleagues with the support of the PPG. Estimates of RR are susceptible to error if they take place outside of the previously determined frequency spectrum. Wavelet functions, an innovative method, are used to calculate the relative risk (RR) derived from PPG by the authors of the study [21]. Recent studies have shown that the Hilbert vibration decomposition (HVD) is an effective method for analyzing nonstationary signals [22]. HVD has been used in a broad number of applications relating to the processing of biological signals, one of which is the processing of cardiovascular signals. The elimination of baseline wander from electrocardiograms (ECGs) and the determination of respiratory rate from ECGs are both included in this category of procedures.

A prior study found that artifacts and modest perfusion changes have a substantial influence on the accuracy of HR estimate when PPG signals are filtered in a certain frequency band [23]. This finding was made by the researchers of the previous study. PPG epochs that were at least 30 seconds long were utilized in a number of validation procedures; nonetheless, shorter recordings are preferable for utilization in clinical applications [24]. In the future, further study will be required since the data length of the PPG signal is so short. In order to accomplish precise and trustworthy HR calculation, this will be necessary. In the field of signal processing, the processing of nonstationary signals is accomplished by the use of a nonrecursive approach known as “variational mode decomposition” (VMD) [25]. The VMD algorithm is an intrinsic nonrecursive procedure that does not create any output whatsoever. This decomposition approach has a wide range of potential applications; some examples include monitoring for sleep apnea and seismological time-frequency analysis, as well as voice signal identification. In the empirical wavelet transform, some examples of limitations include recursive shifting, an inability to cope with noise, hard band constraints (wavelet approaches), and specified filter bank borders [26]. An attempt is made to analyze the robustness of different test signals with cryptographic techniques. To resist various attacks, Empirical Mode Decomposition (EMD) is used. Performance evaluation for image watermarking includes robustness, imperceptibility, watermark capacity, and security. The nonrecursive VMD approach was presented by Dragomiretskiy et al. in 2021, and it was then included into the program [27]. When dealing with variational issues, it is essential to make use of approaches that provide the ideal answer, such as mode decomposition. After going through the optimization procedure, you will end up with a mode cluster that has a band limit. VMD is comprised of many Wiener filters that have been merged together [28]. Using this method, it is possible to differentiate between modes that have distinct center frequencies. Wang et al. examined the efficacy of identifying rubbing-caused signals using VMD, EMD, EEMD, and EWT as the four distinct methodologies. They found that VMD was the most effective method. Zhang et al. stated that they were able to effectively recover the rolling bearing signal from a multistage centrifugal pump using VMD [29]. This accomplishment was made before. The results of this study [30] suggest that VMD is superior to other approaches in terms of the number of characteristics it can extract. Tang et al. created an optimization index that was the ratio of the energy that was left over to the energy that was present in the original signal [31]. In this instance, it was decided when the ratio dropped below a certain threshold that had been established beforehand. Because the procedures described in [32] do not take into consideration the properties of the signal component, mode mixing is possible as a consequence of these approaches. Both the VMD mode number and the penalty parameter were improved thanks to the authors' efforts. Although it is possible to achieve the value that is needed for the parameter, this technique is inefficient. The idea came from Susanta Haldar and a few other people [33].

## 3. Proposed Work

**Figure 1:**
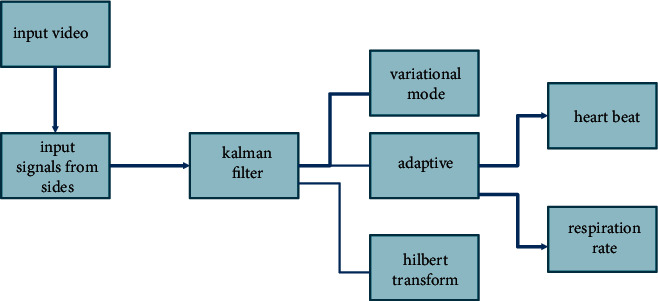
Proposed model architecture.

Because it is used to determine whether or not the heart's activity is healthy, the electrocardiogram, often known as the ECG signal, is the most significant signal in the field of biomedical signal processing. In today's world, the ECG signal processing devices are shrinking in size while simultaneously becoming more compact. That should be equipped with a battery power source so that it may be shrunk down to a more manageable size. A system that is battery driven should have a lower overall power consumption. When this occurs, the circuit has increased functionality and durability. Many different algorithms have been presented over the last few decades in an effort to make the ECG signal processing system as effective as possible [34]. Only by increasing the total number of electrodes did the older algorithms succeed in decreasing the amount of power they used. The procedure of decreasing the number of electrodes is still quite complicated [35]. Communication between the interior of the chip and the outside of the chip is another aspect in the architecture of a SoC that consumes power. Communication with the chip and its peripherals may take place in a variety of modes, including synchronous communication and asynchronous communication, among others. Because the synchronous technique needs a network for clock production and dissemination, the system ends up being more complicated than the asynchronous system. In a system when the clock signal is already there, there must be extra pins for peripheral interface in Figure 1.

In the past, a variety of researches have been carried out for various gating and encoding schemes for the purpose of reducing the unnecessary switching activity of a chip during the process of serial data transmission from on-chip to the outside world or network. These schemes aim to reduce the amount of switching activity that occurs during the transmission. However, the encoding approach either increases the bit size or decreases the power consumption by less than 15 percent. Neither of these options is ideal. Bus-invert coding was suggested as a low-power input-output coding scheme. The development of a data transmission circuit that requires little power is the primary emphasis of this effort. When more components are added to the circuit, the amount of power that is lost as dynamic power due to charging and discharging node capacitances likewise rises [35]. This study applies the approach of coding to the I/O, which has the effect of reducing the activity on the bus. According to reports, the peak power has decreased by fifty percent, while the average power dissipation has decreased by twenty-five percent.

Based on transition inversion, the low power data coding scheme for synchronous serial communication that was suggested is described below. The decrease of power consumption in parallel bus systems is the primary emphasis of this study. This strategy is not appropriate for use with systems in which the transmission takes place in a sequential fashion. The study will be applied to systems like JTAG and SPI, both of which experience significant power loss as a result of data transitions. The work results in a transition decrease that is 39% lower than before. In the research that has been done, there is a technique that has been suggested for the transmission of data in serial mode between the master and the slave. For the purpose of transferring data between the master and the slave, it has been suggested to use a high-speed serial peripheral interface [36].

A regularized channel inversion using dirty paper coding was developed in order to decrease the power offset in MIMO X Channels. The authors based their proposal on a precoding and detection approach. The technology of beam formation has been adapted to be used in this way. The inversion of data and compression of such data are also gaining steam, with the study investigating various canonical sorting permutations in an effort to achieve data compression. When compared to the block sorting method, the move to front strategy indicated above provides superior performance. There is a new approach presented in the literature that has a minimal overhead and uses 34 MSB controlled inversion coding. For the purpose of experimenting with inversion coding, discrete cosine transform and its inverse for a picture are used in practice. There is a 33 percent decrease in the amount of transition activity for DCT data and a 46 percent decrease for IDCT data. The strategy is only useful when the buses are loaded with a significant amount of capacitive components. A method of coding for on-chip flash memory that uses a minimal amount of current was suggested. A sensing amplifier was used in the process that was given the moniker built-in binary coded inversion technique. This approach compared the read current to the reference current. The approach is suggested for use with an ARM Cortex–M3 microprocessor and relies on chip flash memory manufactured using the 180 nm process. A technique known as segmented group inversion coding is one that is based on the inversion-or-not transformation of data that has been specified as being grouped. This technique brings the ratio of ones to zeros down to either one or two, depending on the situation. This strategy is ineffective for computer systems that have less memory and devices that are quicker [37].

It should be noted that even bits are discarded if the number of transitions exceeds 50 percent of the word's total length. For the purpose of error detection, a parity bit has been provided. This approach results in a 7.4 percent reduction in power consumption for the transmission of each bit. An embedded transition inversion (ETI) coding was determined by the phase difference between the data clock and the driving clock [33].

The parallel-to-serial conversion of lines is made easier with the help of the suggested approach, which cuts down on the increased transition bit that occurs during this process.

The article outlines a way that cuts the changeover time by about 30 percent, making it more efficient. The findings for a variety of data patterns have been validated by the work. When employing the optimal spacing and data width, the energy savings are enhanced by a factor of thirty percent. Embedded shift invert transition coding for parallel links was a proposal that was suggested.

### 3.1. Collection of Signal

The sensor interface, which collects ECG data from electrodes attached via the front end signal conditioning unit, is one of the fundamental building components of the system-on-chip (SoC). The filtering block eliminates the undesirable sounds, such as base line drift and physiological disturbances. The processing and decision block is responsible for the extraction of the features, and the communication unit is the one responsible for transmitting the information to the remote unit. The novel encoding unit for the communication block that conveys the information while using less power is the goal of the work that is discussed in this thesis. The switching activity of devices (which occurs when a signal transitions from high to low and vice versa) is the primary driver of power dissipation in a chip. A significant area of focus is the amount of power that is used by the buses throughout the data transmission process [38]. The information is transported all across the chip via the buses, even out to the buffers and into the outside world. In addition, the switching activity of the buffers has a significant impact on the total amount of power used. Therefore, there is the potential to save a significant amount of power by lowering the amount of power that is used by buses and buffers. In addition to that, the length of the individual data packets is another significant aspect related to data.

The throughput, the delay, and the energy usage are all determined by the data length. On-chip design may often play a significant part in multicore architectures, which is necessary in order to alleviate the issues that are caused by long data networks.

**Figure 2:**
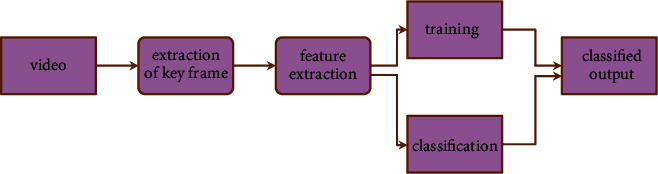
Architecture for video segmentation framework.

They eventually form a component of the architecture of the system-on-chip (Kim et al). When the length is increased, the number of header and tail flits will decrease, but the number of null flits will remain the same. This will occur when the individual packet length is increased. There is not much of a shift in the overall amount of no-data flits, and there is also not much of a shift in the architecture's throughput, latency, or energy consumption. The bandwidth and power limits may be significantly reduced by designing a different coding architecture for the communication unit of the SoC as illustrated in Figure 2.

In the form of a system-on-a-chip architecture, portable biomedical devices include fundamental components such as sensors, a power management circuit, a digital signal processor, inbuilt flash memories, a transmitter, and input and output devices. Other fundamental components include a power management circuit, a digital signal processor, and inbuilt flash memories. They make use of any and all kinds of communication that are available to them. The sensors act as an interface medium to collect real-time patient data, which is then sent to the digital processing unit so that a choice may be made based on the information that was collected. Portable biomedical devices may have a wide variety of sensors, some of which are designed to monitor vital signs such as the heart rate, insulin level, and pulse rate, while others monitor other parameters.

A microprocessor includes programming memory, which holds calculating formulas, and an input/output (I/O) controller, which acts differently depending on how the inputs are configured. Both of these memories are connected via a bus. The multiplier, shifter, and adder lookup unit (ALU) circuits are the most important parts of the DSP controller, which is used by the mathematical processing unit. The DSP controller is used to convert digital signals into analog signals.

#### 3.1.1. Extraction of Key Frame for the Shot Abstraction of Video

Consider one video shot *v* of *F* frames, for example, *v*={*n*_1_, *n*_2_,…*n*_*F*_}, and the extraction process of key frames classifies the taken shot videos into *C* clusters, where *C*=*C*_1_, *C*_2_,…*C*_*R*_. The frame-oriented color histogram is used as the feature in this algorithm, hence can be extracted easily and with low risk. The resemblance between the frames n_i and_ n_j_ is found using

(1) $$\begin{array}{c} X\left(n_{i},n_{j}\right)=\sum_{\alpha =1}^{n}\sum_{\beta =1}^{m}\min\left(X_{i}\left(\alpha ,\beta \right),X_{j}\left(\alpha ,\beta \right)\right). \end{array}$$

If the resemblance value possesses more means, the identical frames are more similar when considering the histogram. When a new cluster is added to the group of clusters, then the centroid value is to be calculated first. The key frame is extracted from the sequence of clusters by comparing it with the threshold value, T.

#### 3.1.2. Object Segmentation Using Model-Based Clustering

In this approach, object-based segmentation from the video is extracted using the GMM model. The Gaussian distribution is used because it is highly traceable and the central limit theorem used here guarantees the summing of random variables from the Gaussian distribution. Hence, the performance of GMM is better, as no-data assumption is made possible over here. A probabilistic video-based segmentation is used for extracting the object from the video segments. The probabilistic space determination is made by the abstraction of feature samples from a set of Gaussian mixtures. The estimation of density in GMM is obtained in a semi-parametric mode as the complexity of the data is a deterministic factor and the size of data is a nondeterministic factor.

#### 3.1.3. Feature Extraction

The raw video data which are in the time-space are transformed into multidimensional feature space, in which the feature vectors are provided with a topology for regularization like the patterns of motion, color, and textures of the video information's. The selection of feature is used for identifying the effective features, but somehow it is not possible to extract the whole contents because of dimensionality variation. The effectiveness of the features will be depending on the selection methods and the extraction methods by considering the motion, color, and the texture. Here in this approach, a pixel-wise feature extraction is used which directly extracts the video data using the extraction process. The feature extraction is made for all the pixels in the frames.

#### 3.1.4. Key Frame Refinement

The extraction of key frames is used for facilitating the object-based video segmentation. The clustering results are used for refining the key frames which will make the shot-oriented representation compactible because of GMM. The extraction of key frame is made with the help of threshold value T. This will make the selection of video frames to be efficient and is needed more for object-based representation. After the extraction of key frames, a key frame set S is obtained as *S*={*kn*_1_, *kn*_2_,…*kn*_*r*_}. The frame index is denoted as f (i). The key frames in the set S are partitioned into N regions *Y*_*j*_^*f*(*i*)^,  *j*=1,2,…*G*, where *G* is the total number of GMM components in the overall process.

The distance between the *Y*_*j*_^*f*(*i*)^and*Y*_*j*_^*f*(*i*+1)^ is calculated using

(2) $$\begin{array}{c} D\left(Y_{j}{}^{f\left(i\right)},Y_{j}{}^{f\left(i+1\right)}\right)=\frac{X\left(Y_{j}{}^{f\left(i\right)},Y_{j}{}^{f\left(i+1\right)}\right)}{2}. \end{array}$$

Then, the distance in between the two successive key frames kn_i_ and kn_j_ is calculated using the following mathematical expression:

(3) $$\begin{array}{c} \text{Distance}\left(kn_{i},kn_{i+1}\right)=\sum_{j=1}^{K}D\left(Y_{j}{}^{f\left(i+1\right)},Y_{j}{}^{f\left(i\right)}\right). \end{array}$$

### 3.2. Adaptive Kalman Filter

The information from the raw video signals is segmented to video frames, and the shot video signals are interpolated to 23 frames per second. Then, the normalization process is started from the obtained signal X (t) as

(4) $$\begin{array}{c} X\left(t\right)=\frac{x\left(t\right)-\lambda }{\eta }+\frac{y\left(t\right)-\lambda }{\eta }, \end{array}$$

where *η* and *λ* are the mean and standard deviation of X(t). The Kalman filter is used for smoothing the signal in order to amplify the heart pulse and respiration pulse. Once the attenuation process of the signal is over, then it is subjected to band-pass FIR filter. At last, the heart rate and the respiration rate from the signal using the specific algorithm are used for real-time prediction of the video signals. The robustness and the accuracy are made in control by using Lomb periodogram. The algorithm is shown below in algorithm 1.

### 3.3. Amplification and Smoothing of the Signals

The Kalman filter is used for filtering out the unwanted signals and to retrieve back the original signal. It contains a nonstationary recursive filter for estimating the needed signal from the noisy background.

The Kalman filter is described in steady state with two different stochastic equations.

(5) $$\begin{array}{c} A_{k}=X_{A_{k+1}}+w_{k},\\ B_{k}=Y_{A_{k-1}+\mu_{k}}. \end{array}$$

Here, *A*_*k*_=[*a*_*k*,_*a*_*k*−1_, *a*_*k*−2_]^*T*^and *w*_*k*_=[*w*_*k*_, 0,1]^*T*^; the *A*_k_ is the column vector which represents the signal vector with no motion. The estimated value *B*_k_ is a scalar quantity.

The obtained vector value *μ*_k_ is the state transaction noise, and another value *wk* is the measurement noise. The matrix for *X* is determined with the time step value k-1 in consideration with the absence of the noise, and the values are marked as below

(6) $$\begin{array}{c} X=\left[\begin{array}{ccc} 1 & 1 & 0\\ -1 & 0 & 1\\ 1 & 0 & 0 \end{array}\right],\\ Y=\left[\begin{array}{ccc} 2 & 0 & -1 \end{array}\right]. \end{array}$$

Normally, the Kalman filter consists of two different parts like updating the equations based on time constraints and updating the equations based on the measurements.

### 3.4. For Time Updates, the Equation Might Be as Follows

(7) $$\begin{array}{c} {\bar{A}}_{k}=X_{{\bar{A}}_{k+1}}+w_{k},\\ \rho^{-1}{}_{k}=X\rho_{k-1}X^{T}+R_{\eta -1}. \end{array}$$

For measurement updates, the equation might be

(8) $$\begin{array}{c} \Gamma_{k}=\rho_{k}Y^{T}{\left(Y\rho_{k}Y^{T}+Q_{k-1}\right)}^{-1},\\ {\bar{A}}_{k}={\bar{A}}_{k}+\Gamma_{k}\left(B_{k}-Y{\bar{A}}_{k}\right)+R_{k+1}. \end{array}$$

Here, Γ_k_ is the Kalman gain, and the error covariance estimation is determined with the setting of 3 x 3 matrix for the value *ρ*_k_. Then, the error covariance prediction is made with the value *ρ*^−1^. This could be shown in the matrix as

(9) $$\begin{array}{c} R=\left[\begin{array}{ccc} 0.4 & 1 & 0\\ 0 & 0 & 0\\ 0 & -1 & 0 \end{array}\right]. \end{array}$$

For deriving the constants *X* and Y, the value of *A*_k_ is to be determined with uniform sampling rate. Here, *A*_k_ value is set to be *A*_*k*_=*A*(*t*_*k*_), and the value of *k* = 1, 2,…. The spacing is made constant and given for “t” as Δt and hence got the value *t*_*k*+1_=*t*_*k*_+Δ*t*. While estimating *A*_k+1_, we get

(10) $$\begin{array}{c} A\left(t_{k+1}\right)=A\left(t_{k}+\Delta t\right)=A\left(t_{k}\right)+\Delta t\frac{\partial A}{\partial t}. \end{array}$$

The derivative approximation is expressed as

(11) $$\begin{array}{c} \frac{\partial A}{\partial t}=\frac{A\left(t_{k}\right)-A\left(t_{k-1}\right)}{\Delta t}. \end{array}$$

From the above equations, it is clear that the estimated value *B*_k_ possesses some value which is much lower than the predicted value and the final expression for the filter design is formulated as

(12) $$\begin{array}{c} {\bar{Y}}_{k+1}=A_{k}+\alpha \left(A_{k}-A_{k-1}\right)+\beta \left(B_{k}-B_{k-1}\right). \end{array}$$

The smaller value *α* and *β* shows that the *A*_k+1_ exceeds the value *B*_k+1_ that shows the prediction of heart pulse and respiration pulse is marked amplified.

### 3.5. Modified Adaptive Fourier Decomposition (MAFD)

In this research, the MAFD is supporting the adaptive decomposition of the video frames in the process of prediction of the HARR value. The obtained frames are grouped as F(t) which is made to place in H-space and is given as

(13) $$\begin{array}{c} F\left(t\right)=\sum_{\alpha =0}^{n-1}{Ζ}_{k}e^{j\alpha t}+\sum_{\alpha =n-2}^{\infty }Q_{k}e^{j\alpha t}=\sum_{m=1}^{M}s_{m}\left(t\right)+\Psi_{N}\left(t\right),\sum_{\alpha =0}^{\infty }{\left|Z_{k}\right|}^{2}<\infty , \end{array}$$

where *S*_m_(t) is the series of mono components and Ψ_N_ is the standard remainder.

The MAFD uses the ration system for pertaining the orthogonality process by fixing the functions for determining the HARR value. The main process involved in MAFD is to extract the mono components from the sequence of high component generation to the low component generation. The estimation of the energy relation is done by fixing the corresponding value of the standard remainders Ψ_N_.

(14) $$\begin{array}{c} Q_{n}\left(e^{jt}\right)=\Psi_{n-1}\left(e^{jt}\right)\sum_{j=1}^{n-1}\frac{1-\alpha_{j}e^{jwt}}{e^{jwt}-\alpha_{j}}. \end{array}$$

For achieving the higher convergence rate, the obtained energy value of the standard remainder, Ψ_n_, at all parts of the decomposition level is maintained to be minimum. Hence, the maximum rate of the projection is shown below.

(15) $$\begin{array}{c} Z_{n}=\arg\max\text{imum}{\left\{\langle \psi_{n},\varepsilon_{\left\{z_{n}\right\}}\rangle \right\}}^{2}:z_{n}. \end{array}$$

The MAFD value gets differed from the normal Fourier decomposition models. For the normal frequency analysis, the various signals are decomposed with the help of MAFD which purely depends on the distribution of energy that makes it possible for determining the overall frequency ranges with individual energy considerations.

The application of MAFD is measured by considering the noise-based signal which effectively removes the noises by using the Hilbert transform.

(16) $$\begin{array}{c} H\left\{s_{m}\left(t\right)\right\}=\frac{1}{2\pi }\left\{\int_{\tau =-\infty }^{0}s^{1}{}_{m}\left(t\right)\frac{1}{t-\tau }d\tau +\int_{\tau =0}^{\infty }s^{2}{}_{m}\left(t\right)\frac{1}{t-\tau }d\tau \right\}. \end{array}$$

The analytic representation of the obtained noisy signal is determined as

(17) $$\begin{array}{c} \psi \left(t\right)=s_{m}\left(m\right)+jΗ\left\{s_{m}\left(t\right)\right\}. \end{array}$$

(17) is applied as input to the MAFD. The noise signal is expressed as

(18) $$\begin{array}{c} s_{m}\left(t\right)=\gamma \left(t\right)+\omega \left(t\right). \end{array}$$

### 3.6. Enhanced Hilbert Vibration Decomposition (EHVD)

The EHVD will decompose the nonstationary signals with various mono components along with the sequentially varying signals with suitable frequencies and amplitudes. The amplitude variation of the signal is decomposed by considering the first components of the input signal. The main part of the mixture is obtained with the highly complicated amplitude signals with lower amplitude. The instantaneous frequency is computed with the largest component analyzed and is subtracted with the already extracted mono components from the input signals. Hence, the EHVD decomposing of the signal s(t) is obtained by using the mathematical expression

(19) $$\begin{array}{c} s\left(t\right)=\sum_{k}\alpha_{k}\left(t\right)\cos\left(\int \zeta_{k}\left(t\right)\text{d}t\right)+\sum_{k}\beta_{k}\left(t\right)\cos\left(\int \vartheta_{k}\left(t\right)\text{d}t\right). \end{array}$$

The envelope of the signal is represented as *α*(t) and *β*(t). The EHVD method might use the analytical signal representation of the input signal for computing the amplitude of the envelope from the obtained. It is projected with highly complicated respiratory components for attaining the PPG signal which has lower energy components of EHVD.

### 3.7. Improved Variation Mode Decomposition (IVMD)

The IVMD is a completely inherent and adaptable technique that decomposes a signal into many modes with varying center frequencies, energy, and bandwidth. When synthesizing the incoming signal, each sub-signal has a particular sparsity and a central wavelength with low bandwidth.

Here, the parameters which are used for initializing the process might include with some representation of the nodes. The larger values of the IVMD method are not provided with appropriate value, and it may depend upon the application it is used. The larger value in the IVMD method founds difficulties in estimating the center frequencies in an accurate manner. Here, the obtained PPG and BCG signals are decomposed into its corresponding frequency spectrum values. The decomposition of the noise signal is correlated with the noise signals

## 4. Experimental Results

For validating the performance of the proposed model, a set of experiments are conducted with some real-time video samples.

### 4.1. Data Collection

The video samples are taken from 25 participants (12 females and 13 males). The age range among the participants is ranging from 20 to 40 years. The video signals are collected by manually testing the participants with the HARR monitor. The subjects are asked to assemble in a separate hall during periodic intervals. The hall is equipped with all setups supporting real-time observation. A pulse oximeter is used for tracing out the real heartbeat value, the exact value is obtained using the BCG, and the respiration rate is monitored using the method PPG in addition to manual checking. The data collection is made in a random manner by extracting about 10 frames per second for up to 10 minutes. The subjects are allowed to sit freely for 15 minutes; hence, their head motion and face reaction all are noted.

### 4.2. Analysis

The efficiency of the proposed model is tested with different aspects. Initially, the information from the PPG and BCG is obtained with video information. The video information is converted into various frames using the HVS method. The information regarding the signal conversion is shown in Table 1.

From the total information retrieved (i.e., 22500 seconds of video), only a part is considered for the analysis. Most of the contents are removed by a process of smoothing and refinement. Mostly, the video is taken out in real time, and hence, the noise attack is more in the video and it can be removed with the help of the Kalman filter.

**Figure 3:**
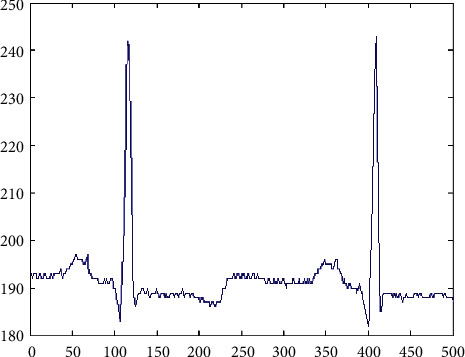
Generation of RGB signal.

Initially, the video signals are preprocessed before feeding into the Kalman filter. Mostly, the videos are taken with the help of cameras with high-resolution pixel representation. After converting the videos into frames, there is a need for checking the synchronization process. The distance between the frames is to be calculated and make sure that the identical distances are to be fixed in between the frames. After then, the signal frames are to be set into various clusters or groups. The obtained RGB signal generated after setting up the groups is shown in Figure 3.

**Figure 4:**
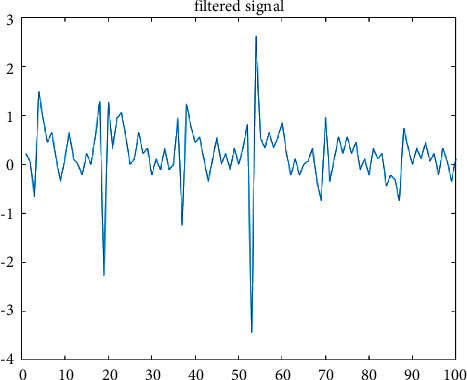
Process of signal detrending.

The groups of RGB signals from the video output are divided into various frames using the suitable segmentation process. Here, the process of detrending the signals is to be needed for estimating the exact RGB value. Since the signals are grouped, there is a need for separation between the frames, so a form of synchronization is needed for combining the original signal with the grouped signal. The detrending process is illustrated in Figure 4.

**Figure 5:**
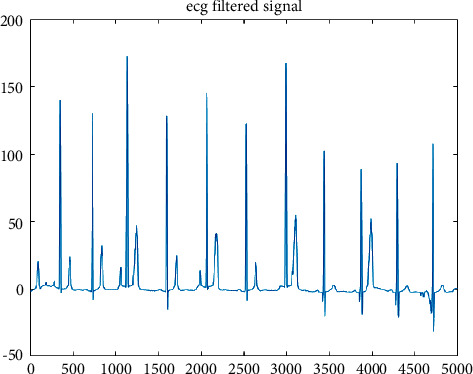
Separation of green signals.

After synchronization, the extraction of green signals from the whole set of frames is needed. The video frame separation is mentioned in another way as green signal separation. For estimating the exact value in separated video frames, the green signal separation supports the process and is illustrated in Figure 5.

From Figure 5, it is clear that the green signals are separated from the whole video sequence. These predicted green signals must possess some errors due to the involvement of noises. In the proposed model, an adaptive Kalman filter is implemented for removing the noises.

**Figure 6:**
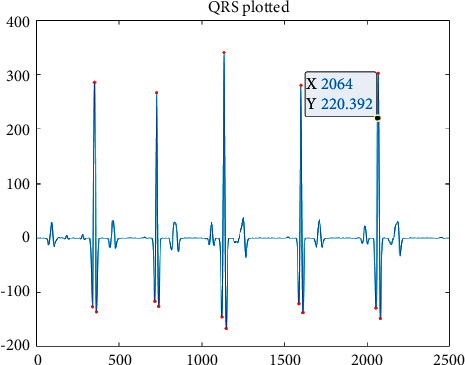
Before Kalman filter.

The noise-included video frames are subjected to an adaptive Kalman filter for further processing. For effective prediction of the HARR value, the removal of noise is mandatory. The signal coming out from the video frames is shown in Figure 6.

**Figure 7:**
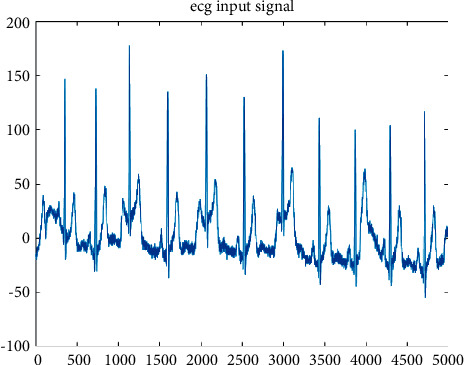
After the implementation of the Kalman filter.

Figure 3 shows the signals retrieved from the video frames are clustered and analyzed. After the implication of the filtering process, the signals get removed with noise and are refined. This is illustrated in Figure 7.

**Figure 8:**
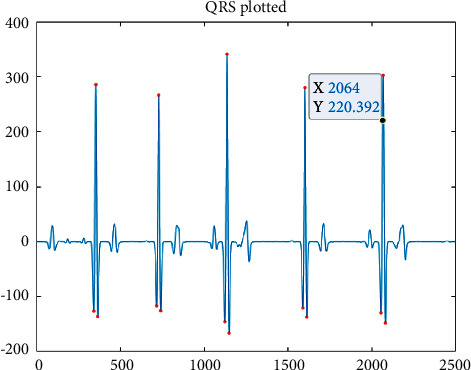
Comparison of Kalman filter.

The change in the peak value shows the effectiveness of the algorithm using the Kalman filter. The variation is predicted with a suitable approach made in the estimation of the true value in association with the Kalman filtered value. The smoothening process is made effective in the determination of the exact value of information without noise. The axis is taken at different intervals within the time and valuable consideration. The exact comparison of the true value and the Kalman filtered value is shown in Figure 8.

**Figure 9:**
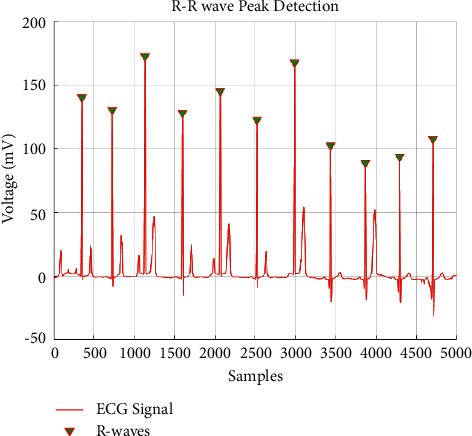
HARR results obtained from EHVD.

The noise-free signals are subjected to the enhanced Hilbert vibration decomposition (EHVD) method, and the result obtained is illustrated in Figure 9.

The parameters are fixed for the values analyzed between the beats per minutes to heart rate and respiration rate. The peak value is to be detected for identification of the peak points where the pulse is so active. The values obtained from the given sources are shown in Table 2.

**Figure 10:**
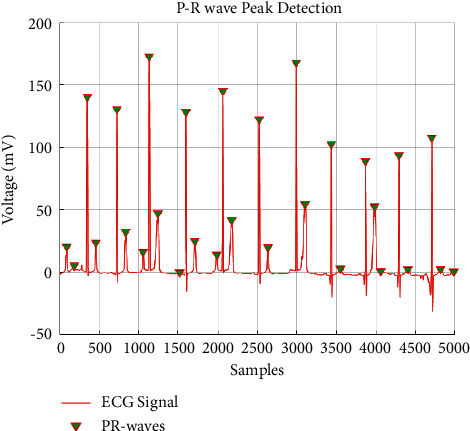
HARR results obtained from IVMD.

Then, the improved variational mode decomposition method is implemented for the determination of the HARR value. The peak value determination shows that the respiration rate and heart beat rate estimation are proved to be more effective in the analysis. This is illustrated in Figure 10.

The estimation is made for the values beats per minutes along with the deterministic values. The total values obtained after the experimentation analysis of IVMD are shown in Table 3.

**Figure 11:**
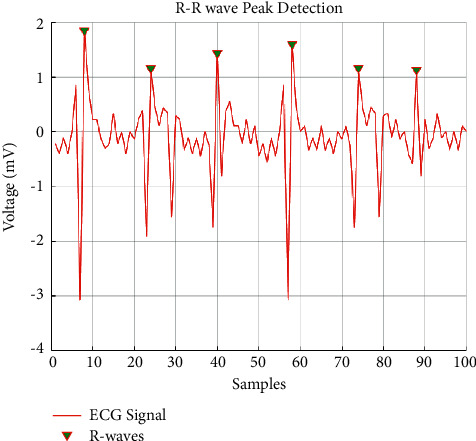
HARR results obtained from MAFD.

The modified adaptive Fourier decomposition is used for the estimation of the heartbeat and the respiration rate. Here, the peak value is identified to be in approximated range in many areas. A form of stability is found in the estimation of signals. The estimation of the HARR value suing MAFD is illustrated in Figure 11.

The value of the HARR after implementing the MAFD method is shown in Table 4.

From the overall analysis held with the estimation of HARR value after the implementation of the three various models like IVMD, MAFD, and EHVD, a small variation was identified. The comparison status of the HARR value along with the three models is shown in Table 5.

## 5. Conclusion

The design of very large-scale integrated circuits (VLSI) provides the foundation for the construction of biomedical systems that can read, evaluate, and make decisions, such as electrocardiograms (ECGs). In the past, academics and research organizations have devised and outlined techniques for the gathering, processing, and sharing of ECG data. The effectiveness of the computational algorithms, the transmission bandwidth, and the number of electrodes employed all contribute to the overall level of complexity. When designing an architecture for a system-on-chip, it is not feasible to use a greater number of electrodes in the acquisition unit, and the computer method has to be as compact as is practically practicable. The portable gadget relies on a battery in order to function, which is of the utmost importance. The incorporation of VLSI design satisfies the criteria for decreased cost, decreased space, and decreased power consumption.

The purpose of this thesis is to propose the creation of an encoding approach for the purpose of communication. The modeled SoC is described in a few chapters, as well as the recommended approach along with comparative analysis. The study is being expanded in the direction of building a processing element called the multiplier design, which provides optimal performance and is suited for operation on SoCs. The design was completed using CMOS technology with a 90 nm process node in order to save both power and space. In order to construct the multiplier, an innovative technique that is based on the lookup table method was used. The design demonstrates superior performance and may be used to carry out the implementation of any functions whose variables include complex or trigonometric expressions. The vast majority of the algorithm for signal processing is devoted to the treatment of exponential and complex functions. Therefore, the suggested multiplier is suitable for usage in the aforementioned contexts [39].

## Figures and Tables

**Algorithm 1:**
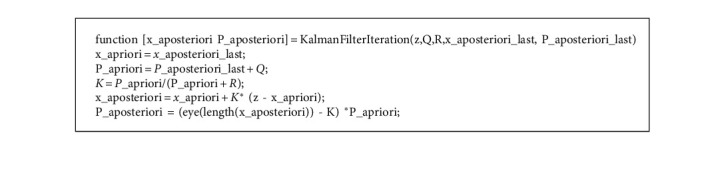
: For Adaptive Kalman Filter.

**Table 1:** Information retrieved from initial observation.

Total number of participants	Total running time of the video (seconds)	Total frames extracted	Total time consumed (seconds)	Frame rate
25	22500	828	5.83 E + 01	1.42 E + 01

**Table 2:** HARR results for EHVD.

*h*	Frame rate	EHVD respiration rate	EHVD heartbeat rate
828	1.42e + 01	4.92e + 00	9.45e + 00

**Table 3:** HARR results for IVMD.

Total frames extracted	Frame rate	IVMD respiration rate	IVMD heartbeat rate
828	1.42e + 01	3.44e + 00	8.27e + 00

**Table 4:** HARR results obtained from MAFD.

Total frames extracted	Frame rate	MAFD Respiration rate	MAFD Heartbeat rate
828	1.42e + 01	2.17e + 00	5.60e + 00

**Table 5:** Comparison of HARR value for various methods.

Methods	Total frames extracted	Frame rate	Respiration rate	Heartbeat rate
MAFD	828	1.42e + 01	2.17e + 00	5.60e + 00
IVMD	828	1.42e + 01	3.44e + 00	8.27e + 00
EHVD	828	1.42e + 01	4.92e + 00	9.45e + 00

From Table 5, it is clear that the respiration rate of the MAFD process is 2.17e + 00, for IVMD it is 3.44e + 00, and for EHVD it is 1.42e + 00. Then, the heartbeat rate is predicted to be 5.60e + 00 for MAFD, 8.27e + 00 for IVMD, and 9.45e + 00 for EHVD. From the obtained values, the EHVD possesses better performance in the estimation of heartbeat rate and respiration rate.

## Data Availability

The corresponding author may provide data to back up the conclusions of this study upon request.
